# Seronegative Chronic Relapsing Inflammatory Optic Neuropathy

**DOI:** 10.7759/cureus.107823

**Published:** 2026-04-27

**Authors:** Keval Thakkar, Chaitanya V Amrutkar

**Affiliations:** 1 Internal Medicine, Willis Knighton Health, Shreveport, USA; 2 Neurology, Willis Knighton Health, Shreveport , USA

**Keywords:** chronic relapsing inflammatory optic neuropathy(crion), immunosuppression, mycophenolate mofetile, optic neuritis, seronegative

## Abstract

Chronic relapsing inflammatory optic neuropathy (CRION) is a rare cause of recurrent optic neuritis characterized by steroid responsiveness and relapse upon treatment withdrawal. Because no definitive diagnostic test exists, the diagnosis is clinical and requires exclusion of more common demyelinating and antibody-mediated disorders.

We describe a 45 year-old woman with a history of psoriasis, photodermatitis, and Hashimoto thyroiditis who presented with four episodes of severe left retro-orbital pain and progressive visual blurring over a three-year period, with the right eye remaining unaffected throughout. Each episode demonstrated left optic nerve enhancement on magnetic resonance imaging and responded promptly to intravenous corticosteroids. Extensive evaluation for multiple sclerosis (MS), neuromyelitis optica spectrum disorder (NMOSD), and myelin oligodendrocyte glycoprotein (MOG) antibody disease was consistently negative. A diagnosis of seronegative CRION was established based on the recurrent steroid-responsive clinical pattern. Long-term immunosuppressive therapy with mycophenolate mofetil, later transitioned to mycophenolic acid due to gastrointestinal intolerance, resulted in clinical stability during follow-up. One additional relapse occurred following a brief three-day interruption of mycophenolate for treatment of cellulitis.

CRION should be considered in patients with recurrent optic neuritis when workup for demyelinating and antibody-mediated conditions is negative. Early recognition and initiation of long-term immunosuppressive therapy are important to reduce relapse frequency and prevent cumulative optic nerve damage.

## Introduction

Optic neuritis is an inflammatory condition of the optic nerve commonly associated with demyelinating diseases such as multiple sclerosis (MS), neuromyelitis optica spectrum disorder (NMOSD), and myelin oligodendrocyte glycoprotein (MOG) antibody disease. In many patients, neuroimaging and laboratory testing help identify the underlying etiology [1-3]. However, some individuals experience recurrent episodes of optic neuritis despite negative diagnostic evaluations for these conditions.

Chronic relapsing inflammatory optic neuropathy (CRION) has been described as a distinct clinical entity characterized by recurrent optic neuritis episodes that show marked responsiveness to corticosteroid therapy and relapse when treatment is withdrawn [4]. Because no single diagnostic test confirms the disorder, the diagnosis is largely clinical and requires exclusion of other demyelinating and inflammatory causes of optic neuropathy [5]. 

CRION is a rare inflammatory optic neuropathy, first formally described by Kidd et al. in 2003, and its exact prevalence remains unknown [4]. The condition is widely believed to be underdiagnosed due to its clinical overlap with more common demyelinating disorders [4,5]. CRION is idiopathic in nature, though an association with systemic autoimmune diseases has been reported in a subset of patients [4,5]. The diagnosis is based on clinical criteria that include recurrent painful attacks of optic neuritis, marked steroid responsiveness, relapse upon steroid withdrawal or tapering, optic nerve enhancement on MRI, and exclusion of other demyelinating and antibody-mediated disorders [4,5].

Recognition of CRION is important because repeated inflammatory episodes may lead to progressive optic nerve injury and permanent visual impairment if not appropriately treated [4,5]. Early initiation of long-term immunosuppressive therapy has been reported to reduce relapse frequency and help preserve visual function in affected patients [6]. We present a case of recurrent, seronegative optic neuritis with four episodes over three years, most consistent with CRION.

## Case presentation

A 45-year-old right-handed woman with a medical history of psoriasis, photodermatitis, and Hashimoto thyroiditis presented with recurrent episodes of severe left retro-orbital pain associated with progressive visual blurring in the left eye. She had no family history of neurologic diseases and reported no tobacco, alcohol, or caffeine use. She had been on hydroxychloroquine for her autoimmune conditions prior to presentation.

Her initial presentation was approximately three years ago, when she developed severe pain behind the left eye radiating to the ipsilateral occipital and posterior cervical regions. The pain worsened with eye movement. Within the following two weeks, she noticed floaters and progressive blurring of vision in the left eye, while the right eye remained unaffected.

**Figure 1:**
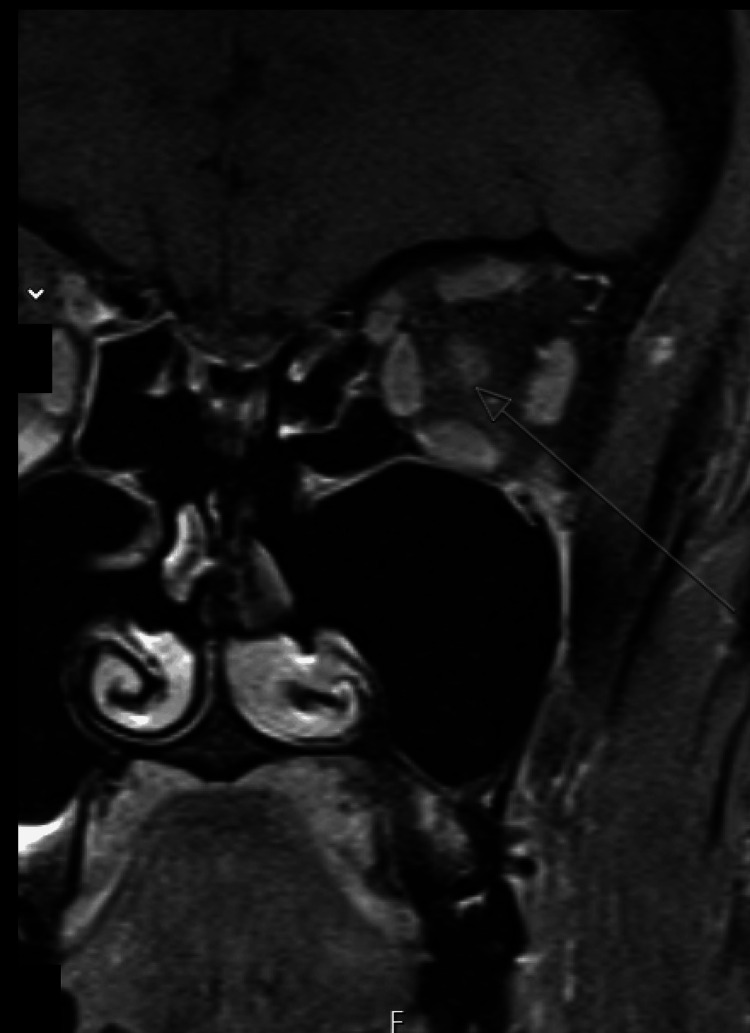
Coronal T1-weighted fat-saturated post-contrast MRI of the orbits demonstrating focal gadolinium enhancement of the orbital segment of the left optic nerve (arrow), consistent with active inflammatory optic neuritis.

MRI of the brain and orbits demonstrated abnormal signal and contrast enhancement involving the left optic nerve, consistent with optic neuritis (Figure 1). A small, non-enhancing, periventricular white matter hyperintensity adjacent to the atrium of the left lateral ventricle was also observed. Lumbar puncture showed normal glucose and protein levels with one nucleated cell and no oligoclonal bands. Infectious and autoimmune testing including rapid plasma reagin (RPR), fluorescent treponemal antibody absorption (FTA-ABS) testing, Bartonella antibodies, rheumatoid factor, and cyclic citrullinated peptide antibodies were negative. Antibodies associated with neuromyelitis optica and MOG disease were also negative. Antinuclear antibody (ANA) was positive at 1:320 with a homogeneous pattern, consistent with the patient's underlying autoimmune history. Angiotensin-converting enzyme (ACE) level was normal. The patient was treated with intravenous methylprednisolone 500 mg daily for three days. 

Approximately three months later, she experienced a second stereotyped episode of left retro-orbital pain, reported to be less intense than the initial episode but preceded by several days of significant psychological stress. Visual disturbance progressed to near-complete loss of vision in the left eye lasting approximately one day before she was evaluated. Repeat MRI demonstrated left optic nerve enhancement without development of new white matter lesions. Cerebrospinal fluid analysis was repeated and remained unremarkable with no oligoclonal bands. MOG and aquaporin-4 (AQP-4) antibodies were repeated and remained negative. Treatment with intravenous methylprednisolone 500 mg daily for three days resulted in near-complete improvement, with a minor residual visual deficit noted thereafter.

Approximately five months after the second episode, she developed a third attack of left optic neuritis and was admitted through the emergency department. MRI demonstrated edema and enhancement involving the orbital and pre-chiasmatic segments of the left optic nerve. Imaging of the cervical and thoracic spine did not demonstrate evidence of demyelinating disease. Brain imaging showed only a few small, nonspecific, subcortical and periventricular white-matter hyperintensities.

Given the recurrent and steroid-responsive pattern of optic neuritis in the absence of diagnostic findings for MS, NMOSD, or MOG antibody disease, a diagnosis of CRION was considered. Long-term immunosuppressive therapy was initiated with mycophenolate mofetil. Due to gastrointestinal intolerance, the medication was later transitioned to mycophenolic acid. Vision recovery following this episode was notably incomplete, with the patient reporting approximately 80% recovery compared to prior baseline.

Several months into treatment, the patient developed cellulitis requiring antibiotic therapy. Her treating physician advised temporary discontinuation of mycophenolate for approximately three days. Shortly after this brief interruption, she developed a fourth episode characterized by deep-seated left retro-orbital pain along with early changes in vision. She was admitted and treated with intravenous methylprednisolone 500 mg daily for two to three days with near-complete resolution of symptoms. Mycophenolate was promptly resumed following resolution of the cellulitis.

**Figure 2:**
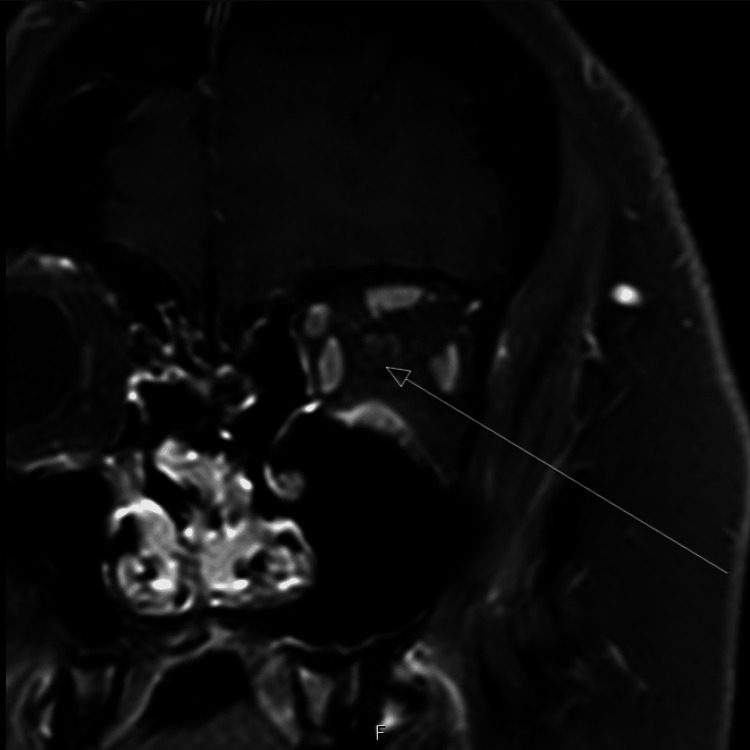
Coronal T1-weighted fat-saturated post-contrast MRI of the orbits obtained during follow-up, demonstrating complete resolution of previously noted left optic nerve enhancement (arrow), indicating response to immunosuppressive therapy and disease stability.

Over the subsequent follow-up period, repeat MRI of the brain and orbits demonstrated stable findings without new lesions or persistent optic nerve enhancement, as seen on follow-up imaging obtained in August 2024 (Figure 2). The patient remained clinically stable on mycophenolic acid with no further relapses. Formal visual acuity, color vision, and visual field testing were not systematically documented at each episode, which represents a limitation of this report. The neurological examination at follow-up was grossly benign with no focal deficits.

## Discussion

CRION is an uncommon but important cause of recurrent optic neuritis. The disorder was first described in patients who developed repeated episodes of optic nerve inflammation that responded dramatically to corticosteroid therapy but recurred when treatment was tapered or discontinued [4]. Because there is no definitive diagnostic test, the diagnosis depends on recognizing the characteristic clinical pattern and excluding other inflammatory and demyelinating causes of optic neuropathy [5].

Several clinical features help differentiate CRION from more common causes of optic neuritis. Patients frequently present with severe retro-orbital pain followed by progressive visual disturbance. MRI often demonstrates optic nerve enhancement during acute attacks [5]. Cerebrospinal fluid studies are typically normal, and oligoclonal bands are generally absent. It should be noted that oligoclonal bands are not specific to MS and may be seen in other inflammatory conditions; their absence helps support a non-MS etiology but should be interpreted in clinical context [2,5].

A critical step in evaluation is the exclusion of NMOSD and MOG antibody disease, both of which can present with recurrent optic neuritis and cause significant visual impairment [2,3]. Of particular relevance are some patients who initially meet diagnostic criteria for CRION but may later test positive for MOG antibodies, underscoring the importance of repeat serologic testing during follow-up [7]. Although antibody testing is an important diagnostic tool, serologic markers may be negative early in the disease course. Treatment strategies can differ substantially depending on the underlying diagnosis, making accurate serologic classification important [2,3,8]. In contrast, patients with CRION are typically seronegative and often demonstrate disease confined primarily to the optic nerve [5].

The hallmark feature of CRION is a marked response to corticosteroid therapy with relapse when steroids are tapered or discontinued [4]. Because repeated attacks may lead to cumulative optic nerve damage, long-term immunosuppressive therapy is frequently required [6,9]. Several agents have been reported in the literature, including azathioprine, methotrexate, mycophenolate, intravenous immunoglobulin, and rituximab [6,10]. Mycophenolate has been used successfully in multiple series due to its relatively favorable safety profile and effectiveness in reducing relapse frequency.

In the present case, the patient experienced four episodes of unilateral optic neuritis over a three-year period, each responding rapidly to corticosteroid therapy. The consistently unilateral pattern is consistent with the published literature, in which unilateral involvement is reported in the majority of CRION cases, though bilateral episodes can occur either simultaneously or sequentially [4,5]. The severity of vision loss varied across episodes, with near-complete vision loss during the second episode and only approximately 80% vision recovery following the third episode, suggesting some degree of cumulative optic nerve injury despite treatment. A brief three-day interruption of mycophenolate was sufficient to trigger a fourth relapse, highlighting how precarious the immunosuppressive balance is in this condition. The patient's coexisting psoriasis, photodermatitis, Hashimoto thyroiditis, and positive ANA titer at 1:320 suggest a broader state of systemic immune dysregulation, consistent with reported associations between CRION and autoimmune conditions [4,5]. Initiation of long-term immunosuppressive therapy resulted in sustained clinical stability during follow-up, with resolution of optic nerve enhancement on repeat imaging.

## Conclusions

CRION should be considered in patients with recurrent optic neuritis when evaluation for more common demyelinating disorders remains negative. In this case, four episodes of left-sided optic neuritis over three years, each responding promptly to intravenous corticosteroids and recurring upon treatment withdrawal, were most consistent with CRION.

A brief three-day interruption of maintenance immunosuppression was sufficient to provoke relapse, underscoring the steroid and immunosuppression dependence characteristic of this condition. The trajectory of vision recovery with diminishing recovery after successive episodes reinforces the need for early diagnosis and uninterrupted maintenance therapy. In this patient, long-term immunosuppressive therapy was associated with sustained clinical stability and resolution of optic nerve enhancement on follow-up imaging. Careful longitudinal follow-up, including repeat serologic testing, is essential to confirm the diagnosis and adjust management as needed.
